# Rasch analysis, reliability, and validity of the Turkish version of the Lumbar Spine Instability Questionnaire

**DOI:** 10.55730/1300-0144.5677

**Published:** 2023-03-23

**Authors:** Erkan EROL, Umut APAYDIN, Ramazan YILDIZ, Ayşe YILDIZ, Sinem ERTURAN, Derya GÖKMEN, Özlem AKKOYUN SERT, Bayram Sönmez ÜNÜVAR, Hasan GERÇEK, Bülent ELBASAN

**Affiliations:** 1 Department of Physiotherapy and Rehabilitation, Faculty of Health Sciences, Tokat Gaziosmanpaşa University, Tokat, Turkiye; 2 Department of Physiotherapy and Rehabilitation, Faculty of Health Sciences, Karadeniz Technical University, Trabzon, Turkiye; 3 Department of Physiotherapy and Rehabilitation, Faculty of Health Sciences, Erzurum Technical University, Erzurum, Turkiye; 4 Department of Physiotherapy and Rehabilitation, Faculty of Health Sciences, Gazi University, Ankara, Turkiye; 5 Department of Biostatistics, Ankara University Faculty of Medicine, Ankara, Turkiye; 6 Department of Physiotherapy and Rehabilitation, School of Health Sciences, KTO Karatay University, Konya, Turkiye; 7 Department of Physiotherapy and Rehabilitation, Vocational School of Health Services, KTO Karatay University, Konya, Turkiye

**Keywords:** Low back pain, cross-cultural adaptation, back instability, Rasch model, validity

## Abstract

**Background/aim:**

Lumbar instability is an important condition that can be seen frequently in people with low back pain, affecting both the progression and the choice of appropriate exercise. The Lumbar Spine Instability Questionnaire (LSIQ) is a simple and low-cost tool for evaluating disturbed back stability in people with low back pain. The aim of this study is to develop the Turkish version of the LSIQ (LSIQ-T) and to evaluate its psychometric properties using the Rasch model.

**Materials and methods:**

One hundred participants with chronic low back pain completed the LSIQ-T. The LSIQ-T was repeated for 30 participants after 1 week to establish its test–retest reliability. While internal and external construct validity were investigated using Rasch analysis and the Spearman correlation coefficient, respectively, reliability was evaluated in terms of internal consistency by Cronbach’s alpha and the Person Separation Index (PSI).

**Results:**

All items of the LSIQ-T were found to fit the Rasch model (chi-square: 34.07 (df = 15), p = 0.0033). The internal construct validity was good, the overall mean item fit residual was 0 (SD: 0.765), and the mean person fit residual was 0.322 (SD: 1.123). Internal consistency reliability was low with a PSI of 0.63 although Cronbach’s alpha was acceptable (0.68). When the test–retest reliability was examined via differential item functioning (DIF) by time, none of the items showed DIF.

**Conclusion:**

The LSIQ-T is a valid unidimensional scale for the Turkish population. Although the LSIQ-T had low internal consistency, it demonstrated unidimensionality and is appropriate for use. Therefore, the LSIQ-T can be used in clinical practice and scientific research.

## 1. Introduction

Low back pain (LBP) is a very common symptom for people of all ages. It can be seen in all countries ranging from high-income to middle-income and low-income and in all age groups from children to the elderly population [1]. A person may experience LBP of 50%–70% throughout his or her life and the prevalence of LBP pain is approximately 18% [2]. Many cases of chronic LBP (CLBP) are of mechanical origin and such LBP cases can also be called clinical spinal instability [3]. Although there is some debate about its definition, clinical spinal instability is generally defined as the loss of the ability of the spine to maintain its proper movement under physiological loads [4]. It is reported that the prevalence of lumbar instability in patients with CLBP ranges from 12% to 57%. In addition, after discectomy surgery, lumbar instability was observed in approximately 12% to 22% of patients at 3 and 5 years of follow-up, respectively [5].

The task of the spinal stabilization system is to provide sufficient stability to the spine to meet constantly changing demands due to changes in the spinal posture and static and dynamic loads. This stabilization is provided by the passive ligamentous system, active musculotendinous system, and neural control system. The problems that occur in these systems, which are essential for stabilization, prepare grounds for spinal instability. Instability in any segment of the spine causes muscle spasm, vulnerability to injury, fatigue, pain, and loss of function [6,7]. For this reason, it is very important to diagnose instability problems in the early period and to conduct appropriate interventions. The diagnosis of clinical spinal instability is controversial and it is noteworthy that most clinical tests used to detect clinical spinal instability have not been validated [8–10]. In a Delphi study to provide consensus on common subjective and objective symptoms associated with clinical instability of the spine, a consensus list of the common characteristics of lumbar clinical instability was established [10]. Based on this consensus list, the 15-point Lumbar Spine Instability Questionnaire (LSIQ) was developed [11,12].

Macedo et al. used the LSIQ for the first time in their study that compared motor control exercises and graded activities in patients with CLBP [12]. The psychometric properties of the LSIQ were not fully tested in that study. Comprehensive evaluations of the questionnaire as a measurement tool are required before the LSIQ can be widely recommended. The psychometric properties of the English, Brazilian Portuguese, Swedish, and Thai versions of the LSIQ were found to be acceptable [5,12–14]. The aim of the present study was to develop a Turkish version of the Lumbar Spine Instability Questionnaire (LSIQ-T) and evaluate its comprehensive psychometric properties using confirmatory factor analysis.

## 2. Materials and methods

### 2.1. Participants

This study was approved by the Gazi University Clinical Research Ethics Committee. Participants were considered eligible if they had experienced LBP for at least 3 months, were aged between 18 and 65 years, and were able to read and speak the Turkish language. They were excluded if they met any of the following criteria: specific causes of LBP (fracture, disc herniation, lumbar stenosis, spinal deformity, or spondylolisthesis), nonmechanical causes of LBP (systemic illness, such as tumors or rheumatological diseases), history of back surgery in the prior 12 months, and conservative treatment in the prior 3 months. One hundred participants with LBP completed the LSIQ-T. Thirty of the 100 patients with CLBP who participated in the study were randomly selected for the test–retest reliability analysis and the LSIQ-T was administered again after 1 week. All patients received written information and signed an informed consent form.

### 2.2. Procedure

Prior to the study, permission was received from L.G. Macedo, who developed the LSIQ. First the translation and the cultural adaptation of the LSIQ were completed considering the stages proposed by Beaton et al. [15]. The original questionnaire was translated from English to Turkish by two native speakers of Turkish. A single version was produced after discussion and consensus between the two translators. Two other translators back-translated the synthesized version of the LSIQ into the original English language and this questionnaire was compared with the original version. Finally, L.G. Macedo, the developer of the LSIQ, approved the final version in the Turkish language. In the pretest phase, 20 individuals with LBP were evaluated for the understanding of the items and words and were asked to complete their responses [15,16]. The comprehensibility of the questionnaire was scored with a “yes” or “no” answer. When replying “no” to the comprehensibility, the participants were asked to explain which items were not comprehensible and to clarify the reasons. According to the results of the pretesting phase, no changes were made to the prefinal version of the LSIQ-T (Table 1).

Functional disability was evaluated using the Turkish version of the Roland–Morris Disability Questionnaire (RMDQ) [17]. Kinesiophobia was assessed using the Turkish version of the Tampa Scale of Kinesiophobia (TSK) [18] and depression was measured with the Beck Depression Inventory (BDI) [19].

### 2.3. Statistical analysis

The internal construct validity of the LSIQ-T was examined using the Rasch measurement model [20]. Internal construct validity in the current study was assessed by the fit of the data to the Rasch dichotomous model. Rasch analysis includes the following sequential steps [21]:

*1. Deletion of misfitting items*

*2. Reanalysis for the overall model and individual item fit*

Fit was determined by a number of fit statistics. At the scale level, summary fit statistics included item and person residuals that, with perfect fit, would have a mean of 0 and a standard deviation of 1. The chi-square interaction fit statistic should be nonsignificant to show a lack of deviation from model expectations. At the individual item level, fit residuals should be between −2.5 and 2.5, and chi-square statistics should be nonsignificant (>0.05 Bonferroni adjusted).

*3. Examination of differential item functioning (DIF) for sex, age, body mass index (BMI), employment status, duration of pain, location of pain, visual analog scale (VAS) score at rest (0–100), and VAS score during activity (0–100)*

DIF, which was examined here for sex (male/female), age (≤34/>34), BMI (≤25.80/>25.80), employment status (employed/unemployed), duration of pain (<12 months/≥12 months), location of pain (only low back/low back and leg), VAS score at rest (≤34.5/>34.5), and VAS score during activity (≤52/>52), should show nonsignificant differences between groups (Bonferroni adjusted).

*4. Examination of local dependency*

The assumption of local independence states that there should be no residual correlation between items once the relevant trait has been extracted. This can be defined as response or trait dependency [22].

The external construct validity of the LSIQ-T was assessed by testing for the expected associations of the Rasch-transformed LSIQ-T score with the RMDQ, TSK, and BDI through the process of convergent construct validity. The degree of associations with these outcome measures was analyzed by Spearman correlation coefficients.

The reliability of the LSIQ-T was examined by internal consistency. An estimate of the internal consistency reliability of the LSIQ-T was tested with the Person Separation Index (PSI) [23], which is equivalent to Cronbach’s alpha [24] but has a linear transformation from the Rasch model. For the test–retest reliability of the LSIQ-T, DIF was assessed to verify the invariance of the item difficulty hierarchy across the first and second assessments (DIF by time). Data were analyzed using RUMM2020, a Rasch model computer program [25].

## 3. Results

The demographic and clinical data of the participants are shown in Table 2.

### 3.1. Rasch analysis

When the 15 items were subjected to Rasch analysis, all items were found to fit the model given a Bonferroni adjustment fit level of 0.0033 (Table 3). The overall mean item fit residual was 0 (SD: 0.765) and the mean person fit residual was 0.322 (SD: 1.123). Item–trait interaction was nonsignificant, supporting the invariance of the items (chi-square: 34.07 (df = 15), p = 0.0033). Cronbach’s alpha and the PSI were 0.68 and 0.63, respectively, indicating that the scale had an acceptable Cronbach’s alpha value (>0.6) but low internal consistency according to the PSI (<0.7). As there were no items showing DIF by time, it could be concluded that the LSIQ-T is a reliable scale in terms of test–retest reliability.

When DIF was tested for the factors mentioned above in Section 2, Item 2 and Item 12 showed DIF by age. While the probability of “feeling the need to frequently pop my back to reduce the pain” was high for people with ages of ≤34, that of “getting temporary relief with back brace or corset” was high for people with ages of >34. In order to determine whether this DIF source was substantial or artificial, we created a subtest for these two items and checked whether there were any items showing DIF. However, after this modification, there were no items showing DIF. All 15 items defined a unidimensional scale of clinical instability since there were no significant differences between the observed and expected scores in terms of p-values. When the assumption of local independence was examined, there was no pair of items that had a residual correlation of 0.30 or more.

Overall, the resulting 15-item scale was not particularly well targeted. With a mean (standard deviation) person score of 0.322 (1.123), patients in this study displayed a higher level of clinical instability than the average (standard deviation) level of the item bank, 0 (0.765) (Figure).

### 3.2. External construct validity

When the correlations of the LSIQ-T Rasch-transformed score with the RMDQ, TSK, and BDI were examined, there were statistically significant positive correlations with the RMDQ, BDI, and TSK (p < 0.05) (Table 4).

## 4. Discussion

The aim of this study was to develop a Turkish version of the LSIQ. The LSIQ-T demonstrated a unidimensional structure and acceptable item fit statistics, supporting its use as a measure of lower back instability. The LSIQ-T was found to be a valid, reliable, and unidimensional scale for patients with LBP. Furthermore, the LSIQ-T is currently the only self-report questionnaire in Turkish assessing low-back instability in individuals with LBP.

Rasch analysis was used to evaluate the validity and reliability of the LSIQ-T. Rasch analysis allows the conversion of a total score into a linear score. In this way, we could perform arithmetical computations and parametric statistical analysis in the present study. When the 15 items were subjected to Rasch analysis, all items were found to fit the model given a Bonferroni adjustment fit level of 0.0033. The overall mean item fit residual was 0 (SD: 0.765) and the mean person fit residual was 0.322 (SD: 1.123). Item–trait interaction was nonsignificant, supporting the invariance of the items (chi-square: 34.07 (df = 15), p = 0.0033). It was seen that the LSIQ-T showed the characteristic of unidimensionality. The findings confirmed the unidimensional character of the LSIQ-T in measuring CLBP, which indicated that the questionnaire’s intraclass correlation coefficient was valid for its use in this patient group.

Cronbach’s alpha coefficient of a scale provides an internal consistency assessment, with values between 0.6 and 0.8 considered to be acceptable [26]. Cronbach’s alpha coefficient was reported as 0.818 in a previous Turkish-version study of the LSIQ [27]. However, in our study, Cronbach’s alpha coefficient was found as 0.68, but the PSI was poor (0.63), indicating that the ability of the scale to differentiate between two groups of patients is low. Macedo et al., the original developers of the LSIQ, performed Rasch analysis of the LSIQ in a more recent study and reported Cronbach’s alpha coefficient (0.69) and PSI (0.64) values similar to those of our study [28]. Similar to that recently published Rasch analysis of the LSIQ, the present study demonstrated that the LSIQ-T is unidimensional but has low ability to differentiate between two groups of patients. In both studies, the low PSI values suggested that more items may be needed to represent individuals with higher levels of instability.

Our study revealed good construct validity as the items did not generally show DIF. Items 2 and 12 showed DIF by age but all remaining items were free of DIF in terms of age, sex, and location. While the probability of “feeling the need to frequently pop my back to reduce the pain” was high for people with ages of ≤34, that of “getting temporary relief with back brace or corset” was high for people with ages of >34. In order to determine whether this DIF source was substantial or artificial, we created a subtest for these two items and checked whether there were any items showing DIF. However, after this modification, there were no items showing DIF. All 15 items defined a unidimensional scale of clinical instability since there were no significant differences between observed and expected scores in terms of p-values. When the assumption of local independence was examined, there was no pair of items with a residual correlation of 0.30 or more.

Regarding construct validity, we found positive correlations with disability, kinesiophobia, and depression. These results are consistent with the previous Turkish version and Brazilian Portuguese version of the LSIQ [13,27].

The development of the LSIQ-T may be useful for a better understanding of the underlying mechanisms of nonspecific LBP. Decreased spinal stabilization is closely related to LBP. It is important to detect spinal instability in individuals with LBP, to determine appropriate treatment programs, and to be successful in treatment. The LSIQ-T may be very useful in clinical practice because it has the benefit of being a simple and easily administered questionnaire.

## 5. Conclusion

The results of this study showed that the LSIQ-T, which assesses self-reported perceptions of spinal instability, is a valid, reliable, and unidimensional scale for the Turkish population. Although the LSIQ-T had low internal consistency, it demonstrated unidimensionality and is appropriate for use. Therefore, the LSIQ-T can be used in clinical practice and in scientific research.

## Figures and Tables

**Figure:**
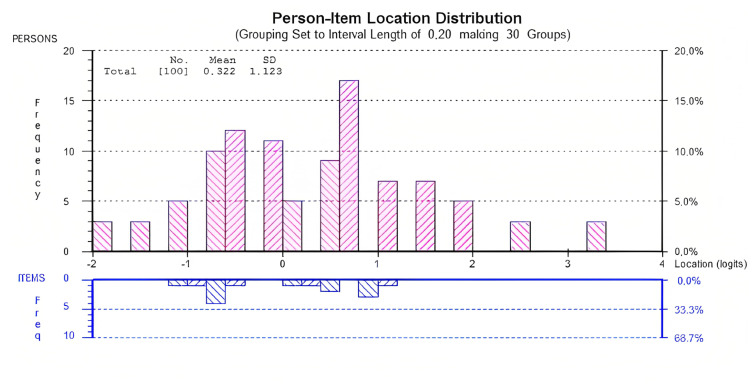
Targeting the LSIQ-T to patients’ clinical instability. SD: Standard deviation.

**Table 1:** Turkish version of the Lumbar Spine Instability Questionnaire.

	EVET	HAYIR
1. Belim tutmayacak, beni taşımayacakmış gibi hissediyorum.		
2. Ağrımı azaltmak için belimi sık sık kütletme ihtiyacı hissediyorum.		
3. Gün içerisinde sık sık ağrım olur.		
4. Geçmişte, dönerken veya eğilirken belim takılmıştı veya kilitlenmişti.		
5. Oturmadan ayağa kalkarken veya ayaktan oturmaya geçerken ağrım olur.		
6. Yatış pozisyonundan oturmaya geçerken eğer doğru şekilde kalkmazsam çok ağrım olur.		
7. Ağrım bazen hızlı, beklenmedik veya hafif hareketlerle artar.		
8. Sırt desteği olmayan bir sandalyede oturmakta zorluk çekiyorum ve sırt desteği ile daha iyi hissediyorum.		
9. Uzun süre hareketsiz kaldığım pozisyonlara katlanamıyorum.		
10. Durumum gittikçe kötüleşiyor gibi geliyor.		
11. Bu sorunu uzun zamandır yaşıyorum.		
12. Bazen kuşak veya korse ile geçici olarak rahatlıyorum.		
13. Birçok durum kas spazmı yaşamama neden olabiliyor.		
14. Bazen ağrım nedeniyle hareket etmekten korkuyorum.		
15. Geçmişte yaşadığım bir travma nedeniyle belim incindi.		

**Table 2:** Demographic and clinical data of the participants.

	Mean ± SD (n = 100)
Age (years)	37.12 ± 15.91
Sex (female) (n/%)	55
BMI (kg/m^2^)	26.12 ± 5.57
LSIQ-T	8.38 ± 3.22
RMDQ	9.09 ± 6.22
TSK	40.52 ± 7.11
BDI	12.88 ± 7.98

Characteristics of the participants are summarized.

LSIQ-T: Turkish version of the Lumbar Spine Instability Questionnaire; BMI: body mass index; RMDQ: Roland–Morris Disability Questionnaire; TSK: Tampa Scale of Kinesiophobia; BDI: Beck Depression Inventory.

**Table 3:** Fit of the LSIQ-T to the Rasch model.

Item	Location	Standard error	Individual item fit residual	Chi-square test statistics	p
LSIQ-T1	0.912	0.232	−2.119	7.853	0.005
LSIQ-T2	0.039	0.224	2.756	1.883	0.170
LSIQ-T3	−0.485	0.232	0.214	1.653	0.199
LSIQ-T4	0.443	0.225	2.540	5.564	0.018
LSIQ-T5	−0.603	0.235	0.704	0.254	0.614
LSIQ-T6	−0.623	0.235	−0.932	2.277	0.131
LSIQ-T7	−0.750	0.239	0.387	0.049	0.824
LSIQ-T8	−0.775	0.240	−0.610	1.068	0.301
LSIQ-T9	−1.006	0.249	−0.235	0.263	0.608
LSIQ-T10	0.958	0.233	−0.746	2.815	0.093
LSIQ-T11	−0.918	0.245	0.151	1.124	0.289
LSIQ-T12	0.867	0.231	−0.167	0.097	0.755
LSIQ-T13	0.547	0.226	−0.778	0.049	0.826
LSIQ-T14	0.363	0.224	−1.530	8.182	0.004
LSIQ-T15	1.031	0.235	1.619	0.944	0.331

LSIQ-T: Turkish version of the Lumbar Spine Instability Questionnaire.

**Table 4:** Correlations between the total score of the LSIQ-T and other scales.

	LSIQ-T Rasch-transformed score	
	r	p
RMDQ	r = 0.613	p < 0.001
BDI	r = 0.292	p = 0.003
TSK	r = 0.344	p < 0.001

Instability questionnaire and clinical variables.

RMDQ: Roland–Morris Disability Questionnaire; TSK: Tampa Scale of Kinesiophobia; BDI: Beck Depression Inventory.
